# Prevalence of hypervirulent *Klebsiella pneumoniae* strains in COVID-19 patients with bacterial co-infections

**DOI:** 10.3389/fmicb.2025.1535893

**Published:** 2025-02-17

**Authors:** Jingfen Zhang, Qiaoyu Li, Jingjing Liu, Fangfang Fan, Yiwei Shi, Xiao Yu

**Affiliations:** ^1^The First Clinical Medical College of Shanxi Medical University, Taiyuan, China; ^2^Academy of Medical Sciences, Department of Occupational Health, Shanxi Medical University, Taiyuan, Shanxi, China; ^3^National Health Commission of the People’s Republic of China (NHC) Key Laboratory of Pneumoconiosis, Shanxi Key Laboratory of Respiratory Diseases, Department of Pulmonary and Critical Care Medicine, The First Hospital of Shanxi Medical University, Taiyuan, Shanxi, China

**Keywords:** hypervirulent *Klebsiella pneumoniae*, COVID-19, bacterial co-infections, sequence type, virulence factor

## Abstract

**Objective:**

A recent alarming report from the World Health Organization highlighted the rapid global spread of a hypervirulent, carbapenem-resistant strain of *Klebsiella pneumoniae*. The COVID-19 pandemic frequently led to bacterial co-infections, with *K. pneumoniae* being a common and highly pathogenic agent. This study aimed to assess KP characteristics via whole-genome sequencing and clarify its molecular epidemiology to guide standardized clinical treatment.

**Methods:**

Our retrospective analysis of clinical data from COVID-19 patients admitted to our hospital between 7 December 2022, and 2 January 2023-following China’s policies changes, which led to a significant influx of patients-identified 17 *K. pneumoniae* isolates from sputum samples with bacterial co-infections. These isolates underwent whole-genome sequencing for ST typing, virulence gene annotation, plasmid profiling, and antimicrobial susceptibility testing.

**Results:**

Of the 17 *K. pneumoniae* isolates, 52.9% were hypermucoviscous. Whole genome sequencing identified eight sequence types (STs), with ST23/KL1 being the most prevalent at 35.3%. Virulence genes were present in 94.1% of strains, including Yersiniabactin (70.6%), Aerobactin (82.3%), and Salmochelin (88.2%). Plasmid analysis revealed common IncHI1B/FIBk or IncFIBk types. All isolates were highly sensitive to antibiotics, except for blaSHV resistance. The 17 patients had a median age of 71 years and significant comorbidities, such as hypertension (64.7%) and diabetes (41.2%).

**Conclusion:**

The ST types and virulence gene profiles indicate that most *K. pneumoniae* strains co-infecting COVID-19 patients are common, high-virulence strains prevalent in the Asia-Pacific region. Our findings suggest that COVID-19 may contribute to the spread of hypervirulent *K. pneumoniae* strains, potentially informing the ongoing WHO epidemic alert.

## Introduction

The COVID-19 pandemic has posed unprecedented challenges to global public health systems, significantly exacerbating the spread of various bacterial pathogens (Fan et al., 2023; Lansbury et al., 2020). A recent alarming report by the World Health Organization (WHO) highlighted the rapid global dissemination of a hypervirulent, carbapenem-resistant strain of *K. pneumoniae*, predominantly the ST23 strain, identified in at least 16 countries and regions since early 2024 (World Health Organization, 2024). Traditionally reported as sporadic or localized clonal outbreaks, the widespread prevalence of this strain under current circumstances remains unexplained. Bacterial co-infections in COVID-19 can lead to severe illness and warrant close attention.

Existing studies have shown that the prevalence of *K. pneumoniae* co-infection with COVID-19 is highest in Asia at 23% (95% CI: 14–35%), followed by Europe at 15% (95% CI: 6–32%), and the Americas at 4% (95% CI: 4–5%). Globally, approximately 17–40% of COVID-19 patients develop bacterial co-infections, with *K. pneumoniae* infections accounting for 19% (ranging from 13 to 28%) of these cases (das Chagas et al., 2024).

Recent studies have shown that *K. pneumoniae* is frequently detected in critically ill COVID-19 patients admitted to the Intensive Care Unit (ICU), as evidenced by respiratory tract and blood samples from these patients (Arcari et al., 2021; Pourajam et al., 2022). The prevalence of multi-drug resistant bacterial infections, particularly those caused by *K. pneumoniae*, is notably associated with the deterioration of patient outcomes, characterized by increased complications, higher morbidity and mortality (Ficik et al., 2023; Navon-Venezia et al., 2017). These findings highlight the crucial role that co-infection with *K. pneumoniae* plays in the progression of COVID-19 to severe conditions.

However, these reports lacked in-depth genomic analysis, focusing primarily on resistance profiles and clinical outcomes. This study aims to use genomic analysis to further investigate the impact of SARS-CoV-2 on the epidemiology of *K. pneumoniae* in our hospital.

## Materials and methods

### Study design and sample collection

This study retrospectively analyzed clinical and microbiological data from COVID-19 patients admitted to our hospital between 7 December 2022, and 2 January 2023, during a period of increased patient influx following a policy shift in COVID-19 prevention. We confirmed KP co-infection in all 17 patients using a comprehensive diagnostic approach that integrated clinical signs, imaging studies (CT or X-ray), blood tests (WBC, NEU%, LYM%, CRP, PCT, etc.), and sputum cultures, ensuring accurate identification. A total of 17 *K. pneumoniae* isolates were obtained from the sputum samples of these patients. Additionally, we extracted clinical data, including demographic characteristics, comorbidities, and laboratory findings, from electronic medical records for further analysis.

### Phenotypic analysis

Phenotypic analysis of the isolates was performed using the string test to identify hypermucoviscosity. A positive result, indicated by a mucoviscous string greater than 5 mm, classified the strain as hypermucoviscous.

### Whole genome sequencing and genomic analysis

Whole genome sequencing (WGS) was conducted to analyze the genomic features of the isolates. Genomic DNA was extracted using a Qiagen DNA Mini Kit, and libraries were prepared using the DNA Library Prep Kit before sequencing on the Illumina HiSeq platform. Sequence assembly was performed with SPAdes, and genome annotation was conducted using Prokka. Sequence types (STs) were determined using the MLST tool, while virulence-associated genes were identified using the BIGSdb-Kp database. Plasmid replicon types were determined with PlasmidFinder, and phylogenetic relationships were analyzed using CSI Phylogeny. Virulence scores were calculated based on the presence of key genes: yersiniabactin, colibactin, and aerobactin. Scores ranged from 0 (no virulence genes detected) to 5 (all three virulence genes present). This scoring system was used to quantify the virulence potential of the isolates. Resistance genes were screened using the ResFinder database.

### Antimicrobial susceptibility testing

Antimicrobial susceptibility testing was performed using the disk diffusion method, following CLSI guidelines. The tested antibiotics included ceftriaxone, meropenem, ciprofloxacin, amikacin, and tigecycline.

### Clinical data analysis

Clinical data were analyzed to identify patient characteristics, including age, sex, comorbidities, and hospitalization duration. Patients were categorized as having either hospital-acquired or community-acquired infections. Statistical analyses were conducted using SPSS, with results presented as medians with interquartile ranges (IQR) or means with standard deviations (SD).

### Ethical approval

Ethical approval for the study was obtained from the Ethics Committee Of Our Hospital (NO.KYLL-2023-091). Informed consent was waived due to the retrospective nature of the research. To protect patient privacy, all genomic data were anonymized and handled in compliance with institutional guidelines and applicable privacy regulations.

## Results and discussion

### Distribution and virulence of *K. pneumoniae* isolates based on phenotypic and genotypic analysis

**FIGURE 1:**
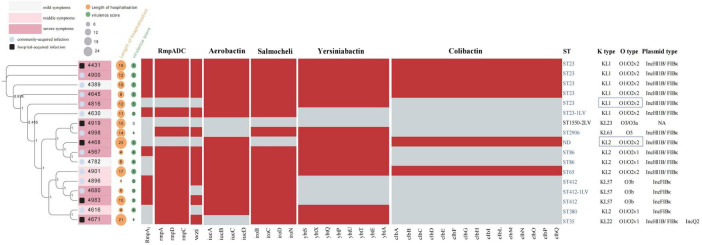
The phylogenetic tree of 17 Kp strains, along with their hospitalization durations, virulence genes, and MLST typing. A total of 17 *K. pneumoniae* (Kp) strains were analyzed, as shown in the left figure, illustrating the evolutionary relationship of Kp strains associated with COVID-19 pneumonia. The intensity of pink within the boxes represents varying degrees of clinical subtypes: deep pink indicates severe conditions, light pink indicates moderate conditions, and light gray represents mild conditions. A black frame around the sample number denotes hospital-acquired infections, while a blue circle indicates community-acquired infections. An orange circle represents the duration of hospitalization, and a green circle reflects virulence gene scores, with the size of each circle proportional to its respective value. Virulence scores are defined as follows: score 0 = no yersiniabactin, colibactin, or aerobactin; 1 = yersiniabactin only; 2 = yersiniabactin and colibactin (or colibactin only); 3 = aerobactin without yersiniabactin or colibactin; 4 = aerobactin with yersiniabactin (no colibactin); and 5 = yersiniabactin, colibactin, and aerobactin. The central heatmap indicates gene presence, with red representing positive genes and gray for negative ones. The right side displays MLST typing and serotypes, with hypervirulent strain ST types highlighted in blue font. Strains with K locus and O locus sequencing issues are shown in the blue boxes on the right.

Phenotypic analysis indicated that 9 of these isolates were hypermucoviscous. Whole genome sequencing identified 8 different sequence types (ST), with 7 of these belonging to highly virulent clonal groups, ST23/KL1 was the most prevalent at 35.3%, followed by ST412/KL57 at 17.6%, and ST86/KL2 at 11.8%. The other isolates corresponded to five different STs: ST35, ST380, ST65, ST2906, and ST1550-2LV, with ST1550-2LV not classified as a highly virulent type (Figure 1). The clustering patterns did not strongly support hospital-based clonal transmission, highlighting the likelihood of community acquisition in most cases. Additionally, ST23/KL1 has been reported to be more prevalent in East Asian regions, which could also explain its dominance in our study.

Upon further analysis of Figure 1, we observed that there was no significant correlation between virulence factor scores and hospitalization duration, clinical signs, and infection modes. This suggests that, while virulence factors such as *rmpA*, aerobactin, and colibactin contribute to the pathogenicity of *K. pneumoniae*, they do not appear to directly impact the length of hospital stay. Instead, factors such as patient comorbidities, immune status, and the severity of underlying COVID-19 infections likely play a more substantial role in determining hospitalization duration. This finding implies that the patients in this cohort may have been carriers of hypermucoviscous *K. pneumoniae*, rather than experiencing severe infections primarily driven by these high-virulence strains. The absence of a strong correlation further supports the notion that co-infection dynamics are multifaceted, influenced by a complex interplay of both bacterial virulence and host-related factors.

### Virulence gene profiles, plasmid types, and antimicrobial susceptibility of *K. pneumoniae* strains

Strains with the same ST carry similar types of high-virulence-associated plasmids (plasmid type: IncHI1B/FIBκ or IncFIBκ) and exhibit comparable virulence gene profiles. The prevalence of virulence genes among these strains is detailed below (Figure 1): yersiniabactin (facilitates iron acquisition, enhancing the survival and virulence of bacteria in iron-limited environments) was detected in 70.6% of the strains (12 strains), with the highest incidence found in *ybt1* (ICE*Kp10*) at 35.2% (6 strains); *ybt4* (plasmid) and *ybt9* (ICE*Kp3*) were each present in 11.7% (2 strains). Salmochelin (glycosylated derivative of enterobactin, another siderophore) had the highest detection rate at 88.2%; aerobactin (siderophore) and *RmpA/RmpA2* (synthesis of the bacterial capsule) were found in 82.3% of strains, and the *wzi* (capsule assembly protein) gene was present in 70.6% of strains. Colibactin (a genotoxin, induces DNA double-strand breaks) displayed the lowest detection rate at 35.3%. The mucoviscosity phenotype-regulating gene, *rmpA*, which is located on the large plasmids of these virulent strains, primarily controls capsule synthesis, resulting in a highly mucoviscous phenotype, observed in 52.9% of the strains. The string test has traditionally identified hvKp strains, but recent studies show that several genetic markers offer high diagnostic accuracy (> 0.95) for detecting hypervirulent strains. These markers include the salmochelin gene (*iroB*), aerobactin gene (*iucA*), and mucoid phenotype regulators (*rmpA* and *rmpA2*). These genes serve as biomarkers to distinguish hypervirulent *K. pneumoniae* (hvKp) from classical strains (Russo et al., 2018). In this study, 16 out of the 17 strains carried one or more of these virulence genes, with only one hospital-acquired strain lacking these genes.

Antimicrobial susceptibility tests revealed that the 17 isolated strains of *K. pneumoniae* were highly sensitive to most antibiotics. Apart from the inherent *bla*_SHV_ gene, no other resistance genes were detected in any of the strains. Our whole-genome SNP analysis revealed significant genetic diversity among the *K. pneumoniae* isolates. Combined with clustering analysis and consideration of isolation sites and collection times, the findings suggest these strains were primarily community-acquired infections, not hospital-based clonal transmissions. Strains with the same ST type also showed no evidence of clonal spread (Supplementary Table 1). The analysis of ST types and virulence gene profiles reveals that the majority of the *K. pneumoniae* strains co-infected with COVID-19 represent common, widespread high-virulence strains prevalent in the Asia-Pacific region.

Traditionally, hypervirulent *K. pneumoniae* strains are mostly sensitive to conventional antibiotics, while clinically prevalent multidrug-resistant strains are generally not hypervirulent, while the strains exhibit high virulence, the limited resistance profiles may reflect an evolutionary trade-off, where acquiring extensive resistance genes could compromise fitness or virulence. Additionally, hypervirulent *K. pneumoniae* strains are traditionally associated with community-acquired infections, where antibiotic selection pressure is lower compared to hospital settings, potentially limiting the acquisition of resistance genes.

### Clinical profiles and emergence of hypervirulent *K. pneumoniae* in COVID-19 patients post policy shift

Clinical data indicate that these 17 patients are elderly and suffer predominantly from underlying health conditions. The median age is 71 years (IQR: 64–78), comprising 9 males and 8 females. The average duration of hospitalization was 11.2 ± 6.7 days. A significant proportion of the patients have comorbidities; 64.7% are diagnosed with hypertension, 41.2% with diabetes, and 35.3% with cardiovascular diseases. These patients were hospitalized due to COVID-19 infections or related complications from their existing health conditions. The clinical data, including imaging and laboratory findings, suggest that the majority of these COVID-19 patients were carriers of high-virulence strains. Furthermore, due to China’s COVID-19 control policies, most hospitals in the country did not admit COVID-19 patients, as these patients were restricted to treatment in designated hospitals. It was only after the policy shift in December 2022 that COVID-19 patients were widely admitted to general hospitals, including ours. This sudden influx provided a unique opportunity to observe bacterial co-infections, including *K. pneumoniae*, under a high patient-load scenario. According to the experience of the clinical microbiology laboratory, there has been a noticeable increase in hypervirulent *K. pneumoniae* infections following the admission of COVID-19 patients. However, standardized testing and reporting for hypervirulent strains remain absent in current clinical microbiology practices, preventing the collection of comprehensive statistical data.

Also, During the COVID-19 pandemic, the World Health Organization observed a noticeable global increase in hypervirulent *K. pneumoniae* strains. This study identified a considerable number of hypervirulent *K. pneumoniae* strains despite the extremely high workload and staff shortages during the study period. Notably, none of these strains belonged to the ST11 hyper-resistant epidemic clone, which is encouraging as patients generally experienced favorable clinical outcomes.

Apart from the patient flow driven by policy changes or factors related to COVID-19 infection, several additional factors may have influenced the prevalence of these strains. Specifically, in healthcare settings, the increase in antimicrobial use was evident in both COVID-19 wards and non-COVID-19 wards. The overuse of antimicrobials, combined with changes in hospital microbiological ecosystems following the large-scale admission of COVID-19 patients, and the increased number of critically ill patients in overcrowded ICUs, likely created an environment more conducive to the survival and dissemination of multidrug-resistant (MDR) and hypervirulent strains. Additionally, altered immune responses in COVID-19 patients may have rendered them more susceptible to infections by hypervirulent bacteria. Moreover, the significant strain on healthcare systems during the pandemic—characterized by surging patient numbers and insufficient resource allocation—may have led to delays in infection control measures and antimicrobial treatments, further facilitating the spread of these hypervirulent strains. Collectively, these factors likely contributed to the post-COVID-19 emergence and prevalence of hypervirulent *K. pneumoniae*.

We acknowledge that the limited data collection timeframe coincided with this policy transition, focusing our observations on a unique and dynamic period. While this context underscores the relevance of our findings, it also limits the ability to observe trends across different COVID-19 waves or under varying healthcare system pressures. This limitation affects the generalizability of our conclusions to other timeframes or regions. To address this, we emphasize the need for future multi-center studies with extended observation periods to comprehensively assess bacterial co-infection trends over time. We plan to collaborate with more hospitals across different regions to investigate regional variations in the prevalence of hypervirulent *K. pneumoniae* strains. Additionally, we aim to analyze factors such as patient demographics, hospital settings, and infection control practices to better understand their impact on strain transmission and clinical outcomes. These studies would help elucidate the broader epidemiology of hypervirulent *K. pneumoniae* strains and their interaction with SARS-CoV-2, particularly under diverse healthcare and pandemic conditions.

## Conclusion

In conclusion, the widespread prevalence of carbapenem-resistant hypervirulent *K. pneumoniae* strains, as highlighted by WHO, is likely predicated on the prior widespread circulation of hypervirulent strains. However, current global and national antimicrobial resistance surveillance systems primarily focus on detecting multidrug resistant strains or isolates from sterile body fluids, with limited attention given to strains isolated from sputum, leaving the true prevalence of hypervirulent strains unclear. It is possible that these hypervirulent strains have acquired carbapenem-resistant plasmids, leading to the observed epidemic. Recently, a study has revealed that during the COVID-19 pandemic, multiple clusters of carbapenem-resistant and hypervirulent *K. pneumoniae* (CR-hvKp) emerged and exhibited significant clonal spread. Post-pandemic, both the prevalence and incidence of CR-hvKp have increased markedly, providing robust support for our hypothesis (Liu et al., 2024). Although our findings are based on a single-center report, they suggest that COVID-19 may have facilitated the widespread dissemination of hypervirulent *K. pneumoniae* strains, potentially explaining the current epidemic. More epidemiological data are needed to confirm this, as these strains have highly mutable genomes and can easily acquire carbapenem-resistant plasmids.

## Data Availability

The datasets presented in this study can be found in online repositories. The names of the repository/repositories and accession number(s) can be found below: https://ngdc.cncb.ac.cn/, CRA019953.
