# Supplementary material for: Prevalence of hypervirulent Klebsiella pneumoniae strains in COVID-19 patients with bacterial co-infections
Source: Front Microbiol. 2025 Feb 17;16:1535893. doi: 10.3389/fmicb.2025.1535893 (PMC11872913; doi:10.3389/fmicb.2025.1535893)
Supplement: Supplementary file 1 [file Table_1.docx]

| Table S 1 Genomic Comparison of the Same Sequence Type (ST) | | | | | | | |
| --- | --- | --- | --- | --- | --- | --- | --- |
| **Sample** | | **Variant-COMPLEX** | **Variant-DEL** | **Variant-INS** | **Variant-MNP** | **Variant-SNP** | **Variant-Total** |
| **ST23** |  |  |  |  |  |  |  |
|  | **4900(4431)** | 17(4.8%) | 20(5.6%) | 13(3.7%) |  | 304(85.9%) | 354 |
|  | **4389(4431)** | 15(6.2%) | 11(4.5%) | 7(2.9%) | 2(0.8%) | 208(85.6%) | 243 |
|  | **4645(4431)** | 37(7.1%) | 13(2.5%) | 15(2.9%) | 3(0.6%) | 453(86.9%) | 521 |
|  | **4816(4431)** | 41(7.9%) | 15(2.9%) | 7(1.4%) |  | 455(87.8%) | 518 |
|  | **4630(4431)** | 137(10.2%) | 24(1.8%) | 20(1.5%) | 14(1.0%) | 1151(85.5%) | 1346 |
| **ST86** |  |  |  |  |  |  |  |
|  | **4782(4567)** | 158(13.0%) | 31(2.5%) | 36(3.0%) | 13(1.1%) | 979(80.4%) | 1217 |
| **ST412** |  |  |  |  |  |  |  |
|  | **4983(4896)** | 1112(9.7%) | 66(0.6%) | 54(0.5%) | 160(1.4%) | 10071(87.9%) | 11463 |
|  | **4680(4896)** | 1008(9.4%) | 64(0.6%) | 49(0.5%) | 142(1.3%) | 9513(88.3%) | 10776 |

Summarize the SNP and INDEL variation information for strains ST23, ST86, and ST412 within each group based on their ST types, including reference samples in parentheses selected from the phylogenetic tree. The table presents detailed numerical values for three ST types (ST23, ST86, and ST412) across various variant categories (including COMPLEX, DEL, INS, NNP, and SNP), along with the percentage contribution of each variant type to the overall variation.
